# Predictors of Booster Engagement Following a Web-Based Brief Intervention for Alcohol Misuse Among National Guard Members: Secondary Analysis of a Randomized Controlled Trial

**DOI:** 10.2196/29397

**Published:** 2021-10-26

**Authors:** Lara N Coughlin, Frederic C Blow, Maureen Walton, Rosalinda V Ignacio, Heather Walters, Lynn Massey, Kristen L Barry, Richard McCormick

**Affiliations:** 1 Addiction Center Department of Psychiatry University of Michigan Ann Arbor, MI United States; 2 Injury Prevention Center University of Michigan Ann Arbor, MI United States; 3 VA Center for Clinical Management Research Department of Veteran Affairs Healthcare System Ann Arbor, MI United States; 4 Department of Biostatistics School of Public Health University of Michigan Ann Arbor, MI United States; 5 Center for Healthcare Research and Policy MetroHealth/Case Western Reserve University Cleveland, OH United States

**Keywords:** alcohol use, National Guard, brief intervention, boosters, engagement

## Abstract

**Background:**

Alcohol misuse is a major health concern among military members. Reserve component members face unique barriers as they live off base with limited access to behavioral health services. Web and app-based brief interventions are a promising means to improve access to treatment for those who misuse alcohol, with the use of booster sessions to enhance effectiveness, solidify gains, and reinforce changes. However, little is known about who will engage in booster sessions.

**Objective:**

This study aims to evaluate booster engagement across booster delivery modalities (Web and Peer) and identify participant-specific factors associated with booster session engagement.

**Methods:**

Following a brief web-based alcohol misuse intervention in National Guard members (N=739), we examined engagement in a series of three booster sessions. Using unadjusted and adjusted models, demographic and clinical characteristics that may serve as predictors of booster session engagement were examined across the 2 arms of the trial with different types of booster sessions: peer-delivered (N=245) and web-delivered (N=246).

**Results:**

Booster session completion was greater for Peer than Web Booster sessions, with 142 (58%) service members in the Peer Booster arm completing all three boosters compared with only 108 (44%) of participants in the Web Booster arm (*χ*^2^_3_=10.3; *P*=.006). In a model in which the 2 groups were combined, socioeconomic factors predicted booster engagement. In separate models, the demographic and clinical predictors of booster engagement varied between the 2 delivery modalities.

**Conclusions:**

The use of peer-delivered boosters, especially among subsets of reserve members at risk of lack of engagement, may foster greater uptake and improve treatment outcomes.

**Trial Registration:**

ClinicalTrials.gov NCT02181283; https://clinicaltrials.gov/ct2/show/NCT02181283

## Introduction

### Background

Alcohol misuse is a major health concern among military members. A Department of Defense Health Related Behaviors Survey of active duty personnel, conducted in 2015-2016, found high rates of hazardous drinking (35%) and binge drinking (30%) [1]. Previous national surveys have found similarly high rates of alcohol misuse [2,3], and these rates were higher among deployed reserve component members, including the National Guard, than among deployed active duty members [4]. Alcohol misuse adversely affects the psychological health and well-being of service members and their families and has a direct impact on their resilience and military readiness [5].

Reserve components have been used heavily during the recent wars, accounting for 28% of all deployments [6]. The approximately 336,000 Army National Guard members nationwide make up 41% of the operational Army forces [7]. They are projected to be an integral part of the fighting force going forward [6]. Maintaining the resilience and readiness of military members, including addressing alcohol misuse, is a particular challenge for reserve component members who do not live and train on military bases where many support services are available, and who spend most of their time in their civilian roles.

Stigma and confidentiality concerns are significant barriers to seeking help with alcohol-related problems in the military [8-10]. Reserve component members face additional unique barriers to seeking help. Unlike active duty members who live on or near military bases where many services are readily available, reserve component members generally attend scheduled training activities only 1 weekend per month, and often live in rural areas remote from their home armory or unit. Web-based and mobile apps can help ameliorate these challenges [11].

The use of computers to screen and deliver brief intervention (BI) has been shown to be effective for a variety of health behaviors, including alcohol use [12,13]. The eHealth interventions for alcohol use seem to be especially useful for older, nonstudent populations [14]. Patients often evaluate computer interactions positively [15,16], and participants may more accurately record sensitive information on computers because of fewer concerns about judgment [17,18].

Few studies of web-based approaches have been reported with military populations, and controlled trials have been hampered by low follow-up rates [19,20]. The Department of Defense is committed to expanding the use of mobile and web-based approaches, but the development and testing of these interventions is needed [21]. A small body of research indicates that the use of booster sessions can enhance effectiveness, solidify gains, and reinforce changes after an initial BI [22-24], including after a computerized intervention, but results are mixed [25-28].

Increasing engagement positively impacts health-related behavior change, including reducing alcohol misuse [29,30], but there is limited understanding of the factors that are associated with engagement [31]. Initial evidence suggests that older age, higher educational attainment, and being female are associated with greater engagement in app- and web-based interventions [31-34], potentially reflecting characteristics that are also broadly associated with greater study adherence.

Despite their potential importance, booster sessions create a number of logistical challenges. They are resource-intensive, and sessions are difficult to deliver when participants are at a distance from their provider. Furthermore, boosters may have limited benefits for some individuals, while being essential for sustained outcomes in others. To date, limited research has sought to identify the characteristics of individuals who are likely to engage in booster sessions following BIs for alcohol and other substance use. In a recent study, Hatch-Maillette et al [35] found that people receiving a BI targeting reduced substance misuse during a visit in the emergency department were more likely to engage in phone-based booster sessions during the month following the BI if they were older, regularly employed, and if they believed substance use was related to the emergency department visit. There is also limited research on the impact of various booster delivery systems on engagement with booster sessions, as measured by the number of booster sessions completed by the participant. Given that in many studies, gains in reducing alcohol misuse dissipate over time, more information on factors associated with booster engagement is needed. Furthermore, the optimal delivery modality for delivery boosters is uncertain.

For example, research indicates that peer support is useful in the treatment of individuals with substance use disorders [36,37]. The use of peer-to-peer interactions has expanded in recent years in Veterans Health Administration facilities and other health care systems and has been widely accepted. The military has recognized the unique value of harnessing the power of peer support and identification with a military peer group to overcome stigma and enhance participation in resilience building and help seeking. For example, the Army's Comprehensive Soldier and Family Fitness Program heavily used unit-based noncommissioned officers as trained trainers who teach and work with soldiers to increase their resilience and overcome stigma for seeking help [38,39]. Numerous *grassroots* peer programs have also been implemented within the military and National Guard, using fellow veterans trained for a range of counseling and support functions. For example, the Buddy-to-Buddy program [40] has been implemented in the Michigan National Guard and in other states but does not focus on alcohol misuse. Peer counselors benefit from the acceptance and trust afforded to men and women who have also served and have the potential to boost adherence to plans created in a web-based intervention; however, research to verify their efficacy for alcohol misuse is needed.

Alternatively, there is also some evidence that eHealth interventions for hazardous alcohol use that include human contact are more effective [13]. A telephone app intervention for alcohol reduction has been modified and tailored for use with veterans receiving treatment in a primary care setting [41], but there has been no research on the impact of eHealth interventions among military or veteran populations as part of the National Guard.

### Objective

In this paper, we examine two modes of booster delivery, web-based and peer-based, to explore modality-specific differences in booster engagement and predictors of booster engagement by delivery modality. Given the paucity of information on boosters, the study is exploratory, and no specific a priori hypotheses have been made. There is strong evidence in the civilian literature on the efficacy of BIs for adults with at-risk or harmful levels of drinking [42-44], but this approach has not been well studied in military populations. Therefore, the aim of this study is to gain a better understanding of booster session engagement among specific populations. This can inform future intervention approaches in two ways. First, by identifying those who are likely to engage with boosters, limited resources can be allocated to those who are likely to use the services. Second, those who are unlikely to engage in booster sessions may constitute a hard-to-reach subpopulation of National Guard service people for whom more intensive engagement approaches are needed.

## Methods

### Study Design

The randomized controlled study, Mission Strong, was approved by the University of Michigan Institutional Review Board and conducted with members of the Michigan Army National Guard attending weekend training drills (NCT02181283). Participants were randomly assigned to the Intervention + Web Booster (N=246) or Intervention + Peer Booster interventions (N=246), or to a control a that did not contain intervention content (N=248).

All intervention participants, regardless of booster modality, initially completed a 30-40 minutes web-based BI based on motivational interviewing, which was tailored for each participant (by sex, deployment history, and baseline drinking pattern) using the FRAMES format [45]: personalized *feedback* (regarding substance use, risk factors), emphasis on *responsibility* for change, *advice*, *menu* of options, *empathic* clinical behaviors, and support of *self-efficacy* regarding making changes. The intervention included a review of participants’ goals and strengths, feedback regarding their present alcohol or drug use patterns and the consequences of their drinking, a decisional balance exercise developing the discrepancy between their alcohol use and ability to meet their goals, an assessment of their readiness to change, and the formulation of a plan with strategies for change. The intervention was designed in a virtual therapist interactive style, with a personally selected military or nonmilitary avatar guiding the participant on his or her *mission* to navigate through the program. The content of the BI protocol was built on existing intervention content in place from previous work by the investigators, but the content was modified to be appropriate within a reserve component military context [46,47].

The overall study included participants who misused alcohol only (N=711) misused only prescription drugs (N=18), and those who misused both alcohol and prescription drugs (N=28). Only participants who misused alcohol alone or alcohol plus prescription drugs were included in the current analyses (N=739). Individuals were eligible to participate if they had an Alcohol Use Disorders Identification Test–Consumption score of 5 or more for men and 4 or more for women, indicating that they met the criteria for at-risk drinking or alcohol misuse [48]. Participants were excluded if they reported receiving substance use treatment in the past 4 months. All participants were provided access to a resource brochure with relevant information for treatment and crisis services. For participants who had an AUDIT [49] score of 19 or more, indicative of a likely alcohol use disorder, the resource brochure was reviewed with the participant with the recommendation to look into treatment options. Following the baseline assessment, participants were randomized using a computer-generated algorithm and stratified by gender and recent alcohol, prescription opiate or sedative misuse into either the web-based intervention plus web administered boosters, the web-based Intervention plus Peer Boosters, or the enhanced usual care (EUC) control arm.

At the end of the BI, participants in the Intervention + Web Booster arm were reminded that they would receive emails once a month for three months, instructing them to log in to complete booster sessions. Those assigned to the Intervention + Peer Booster arm were informed that they would be contacted by peer support veterans at least once a month for three months for follow-up support. If a participant missed a schedule booster, regardless of the arm, contact attempts were made 1-2 times per week (via preferred means of contact, eg, phone, text, email) until the participant completed the booster or the date of the next appointment arrived. Participants were remunerated US $20 for completion of the baseline assessment, US $15 for completing the BI, US $5 for each of the 3 booster sessions, US $35 for the 4-month follow up, US $40 for the 8-month follow-up, and US $45 for the 12-month follow-up.

Sample sizes were determined using power analysis for the primary outcome study. Sample sizes were chosen to allow the comparison of each treatment arm against the usual care arm.

### Booster Types

#### Web Boosters

The Web Booster content mirrored the last three sections of the BI. Messages were tailored based on whether the participant misused only alcohol or both alcohol and prescription medication. Like the BI, boosters were structured as a mission, including different points on a map that the participant had to navigate. They also included video message that provided testimonials that were tailored based on alcohol and prescription opioids as indicated and were based on deployment history (yes or no). Web and Peer Boosters were structured to require approximately 15 minutes to complete, but because they were done independently, no exact data on the time it took to complete them for each participant was available.

#### Peer Boosters

Peer Boosters, a type of person-delivered booster that uses people with similarities to the target population, were delivered by veterans and were conducted over the phone or in person (414/466, 88.8% delivered by phone), lasting approximately 15-20 min. The Peer Boosters had content parallel to that of the Web Boosters, albeit with some differences. At the first booster, peers shared the purpose of the session and asked about the experiences with the first BI session. Survey data were not shared with peers, and peers did not directly ask about the level of alcohol consumption or level of prescription drug use. However, the participants’ individual goals established in the BI were reviewed, which tailored the booster sessions for those who also misused prescription drugs. The booster session meetings started with a discussion of health habits, proceeding to choices, and concluding with the next steps (sections 4-6 of Table 1). The topics discussed at each booster were identical to those of the web sessions, namely finances and reasons for use, physical fitness and mood, and getting places (driving under the influence) and social influences. Table 1 summarizes the contents of the three booster sessions for each treatment arm.

**Table 1:** Web Booster session content.

Booster 1		Booster 2	Booster 3
**Finances and reasons for use**		Physical fitness and mood	Getting places: driving under the influence and social influences
	1. Review past session (strengths, goals, strategies)	1. Review past session (strengths, goals, strategies)	1. Review past session (strengths, goals, strategies)
	2. Current health habits (use and guidelines)	2. Current health habits (use and guidelines)	2. Current health habits (use and guidelines)
	3. Calculator of money spent on alcohol	3. Calculator of alcohol calories	3. Calculator of BAC^b^ + how to get home safely
	4. What could I spend money on instead? How use affects activities and spending?	4. Exercise, mood, and drinking	4. Social influences on drinking
	5. Video of a peer message and strategies (eg, coping, leisure activities, use reduction strategies, safe rides home, pain or stress or sleep management)	N/A^a^	N/A
	6. Summary and plan with one next step	N/A	N/A

^a^N/A: not applicable.

^b^BAC: blood alcohol concentration.

### Measures

Demographic, military, and clinical characteristics were assessed at baseline. Military-specific measures included their military rank and the number of previous deployments, including both international and domestic deployments. Clinical characteristics included past 4-month cannabis or other illicit drug use (cocaine, methamphetamines, hallucinogens, inhalants, heroin, or prescription amphetamines [eg, Ritalin, Adderall]) using the Alcohol, Smoking, and Substance Involvement Screening Test [50]. Alcohol misuse was measured using the 10-item AUDIT [49]. How often the participant drove while drinking in the past 4 months was assessed using a single-item Likert scale ranging from 0 (never) to 4 (10+ times).

Mental health measures included depression, measured using a 9-item depression screen, the Patient Health Questionnaire (PHQ-9) [51]; anxiety, measured using the 7-item General Anxiety Disorder Questionnaire (GAD) [52]; and a history of exposure to trauma, measured using the Mini International Neuropsychiatric Interview (MINI) PTSD module [53].

The current motivation to change alcohol use was measured using a motivation for change ruler scaled from 1 (not ready) to 10 (very ready); the degree of confidence that you can change in the next 4 months was measured with a similar ruler [54]. Motivation for drinking or drug use was assessed using questions from the Drinking Motives Questionnaire-Revised [55]. Drinking Motives Questionnaire-Revised items measuring all 5 subscales were included: Enhancement (eg, drinks or uses drugs to get high, because they like the feeling); coping with anxiety (eg, drinks or uses drugs when anxious); coping with depression (eg, drinks or uses drugs when depressed); conformity (drinks or uses drugs so will not feel left out), and social (eg, what my friends do when get together). Items from the Washington University Risk Behavior Assessment for Club Drugs [56] were used to create an additional subscale assessing motivation to drink or use drugs to obtain physical effects (eg, to function better physically or for pain). The Short Index of Problems (SIP) version of the Drinker Inventory of Consequences Impulsivity Scale was used to measure problems with impulsivity associated with drinking [57].

### Analyses

All analyses were performed using the SAS software (version 9.4, SAS Institute) [58]. General linear modeling (GLM) and chi-square tests were used to examine the unadjusted associations between booster session engagement and follow-up assessment adherence and demographic and clinical characteristics separately for those randomized to Intervention + Peer Booster and intervention + Web Booster arms. Because of the lack of information currently in the literature regarding the effect of booster delivery format on engagement and to stimulate further research in this area, we conducted an exploratory analysis on the univariate relationship between delivery method (Web vs Peer) on booster engagement for variables identified as meaningful based on the previous within-arm analyses. The relationship between the modality of booster delivery (Web vs Peer) and booster session engagement was evaluated using the chi-square test.

Finally, stepwise regression analyses were conducted for each treatment arm separately and for the combined sample from both treatment arms, including the treatment arm as a predictor of booster session engagement. The stepwise regression procedure uses a series of alternating forward and backward selection steps to identify variables to include and maintain the model. The criterion for a variable to be entered and maintained in the model at each step was *P*≤.3, which is the default criterion in SAS and balances the risk of including unnecessary predictors with overly stringent inclusion criteria that can exclude meaningful predictors [59,60]. As such, predictors could be retained in a model because they were below the *P*≤.3 threshold even though they were not statistically significant at the *P*<.05. Stepwise regression was chosen because of the limited previous information to inform the selection of predictors of booster session engagement in the general population and the absence of any information in military populations. Given the limited information to guide theory driven variable selection, the data driven stepwise variable selection approach, and the criteria for inclusion and retention in the stepwise process were preferred for this initial, exploratory study of predictors of booster engagement using demographic and clinical variables in a military sample. Of note, for this exploratory regression analysis, the depression (PHQ>4) and anxiety (GAD>9) scales were dichotomized based on clinical cutoffs indicative of positive screening [51,52]. This was done because these are the cutoffs clinically recommended for mild depression when the PHQ is used as a screening instrument in nonclinical samples, such as our National Guard sample.

## Results

### Overview

The study consort diagram is shown in Multimedia Appendix 1. For the purposes of this study, all analyses focused on the two active treatment arms: Intervention + Peer Booster and intervention + Web Booster. Almost all participants randomized to these two arms completed the BI (227/246, 92.3% for Intervention + Web Booster and 228/245, 93.1% for Intervention + Peer Booster). In the Intervention + Web Booster arm, 56.5% (139/246), 53.7% (132/246), and 48.8% (120/246) completed each specific booster session (ie, booster sessions 1, 2, or 3). In the Intervention + Peer Booster arm, 68.9% (169/245), 63.3% (155/245), and 57.9% (142/245) completed booster 1, 2, or 3, respectively. In the Web Booster arm, 61.4% (151/246) completed at least 1 booster, and 68.9% (169/245) completed at least one booster session in the Peer Booster arm. Table 2 presents booster session engagement by the Web and Peer Booster treatment arms. Of the participants randomized to the Peer Booster arm, 142 (58%) completed all 3 booster sessions compared with only 108 (44%) in the Web Booster arm. In both study arms, of the participants who engaged in any booster sessions, most went on to complete all three. In other words, a relatively small portion of participants who engaged in boosters completed only 1 or 2 boosters. Participants randomized to the Peer Booster delivery format were significantly more likely to engage in boosters than participants in the Web Booster arm (χ^2^_3_=10.3; *P*=.006).

**Table 2:** Number of booster sessions completed by delivery modality.^a^

Booster sessions	Web Booster, n (%)	Peer Booster, n (%)
0	95 (39)	76 (31)
1	19 (8)	14 (6)
2	24 (10)	13 (5)
3	108 (44)	142 (58)

^a^χ^2^_3_=10.3; *P*=.006.

### Unadjusted Outcomes

Tables 3 and 4 present the unadjusted associations for the Web and Peer Booster arms between the number of booster sessions completed and demographic and clinical characteristics. In both treatment arms, those who completed more boosters were significantly more likely to be older. Because of the relatively small number of participants completing either 1 or 2 booster sessions, the participants were combined into a category of those completing either 1 or 2 boosters. In the Web Booster arm, there was a significant relationship between more booster engagement and higher rank, more education, and higher income, as well as trauma history. In the Peer Booster arm, men, people with more education, higher income, deployment history, current employment, married or living together, and experiencing more severe depression or anxiety symptoms were more likely to engage in the booster sessions.

**Table 3:** Unadjusted association between baseline characteristics and Web Booster engagement.

Predictors				Web Boosters																
				0 (n=95)		1 or 2 (n=43)		3 (n=108)		Test statistic (*df*)				*P* value				Effect size^a^		
**Follow-up completion, n (%)**																				
	4 months			31 (17.61)		37 (21.02)		108 (61.36)		*χ*^2^_2_=118.0				<.001				.69		
	8 months			35 (20.00)		34 (19.43)		106 (60.57)		*χ*^2^_2_=94.1				<.001				.62		
	12 months			37 (21.14)		36 (20.57)		102 (58.29)		*χ*^2^_2_=79.8				<.001				.57		
**Baseline characteristics**																				
	Sex (male), n (%)			86 (41.55)		34 (16.07)		87 (42.03)		*χ*^2^_2_=4.7				.09				.14		
	Age, mean (SD)			27.4 (7.5)		27.9 (7.4)		30.5 (7.7)		*F*_1,244_=8.54				.004				.03		
	**Race, n (%)**										*χ*^2^_2_=1.1				.57				.07	
		White	77 (40.10)		34 (17.71)		81 (42.19)													
		Other	18 (33.33)		9 (16.67)		27 (50.00)													
	Hispanic (yes), n (%)			13 (48.15)		3 (11.11)		11 (40.74)		*χ*^2^_2_=1.4				.48				.08		
**Marital status, n (%)**												*χ*^2^_6_=2.4				.87				.07
	Married			25 (33.33)		13 (17.33)		37 (49.33)												
	Living together			16 (38.10)		7 (16.67)		19 (45.24)												
	Widowed, divorced, or separated			10 (35.71)		5 (17.86)		13 (46.43)												
	Never married			44 (43.56)		18 (17.82)		39 (38.61)												
**Highest grade completed, n (%)**												*χ*^2^_4_=26.1				<.001				.23
	High school or less			30 (58.82)		9 (17.65)		12 (23.53)												
	Some college			54 (40.60)		25 (18.80)		54 (40.60)												
	College or more			11 (17.74)		9 (14.52)		42 (67.74)												
**Rank, n (%)**												*χ*^2^4=19.1				<.001				.20
	E1-E4			60 (45.11)		29 (21.80)		44 (33.08)												
	E5-E9			31 (32.98)		14 (14.89)		49 (52.13)												
	WO1-WO5/O1-O9			4 (21.05)		0 (0)		15 (78.95)												
Employed (yes), n (%)				81 (36.99)		40 (18.26)		98 (44.75)		*χ*^2^_2_=2.4				.22				.10		
**Household income (US $), n (%)**												*χ*^2^_6_=19.1				.004				.20
	25,000 or less			29 (46.48)		10 (15.38)		26 (40.00)												
	25,000-50,000			33 (24.49)		12 (16.90)		26 (36.62)												
	50,000 or more			24 (24.49)		19 (19.39)		55 (56.12)												
	Refused			9 (75.00)		2 (16.67)		1 (8.33)												
Ever deployed (yes), n (%)				45 (37.19)		23 (19.01)		53 (43.80)		*χ*^2^_2_=0.4				.80				.04		
Trauma exposure (yes), n (%)				21(26.25)		17 (21.25)		42 (52.50)		*χ*^2^_2_=7.6				.02				.18		
Illicit drug use (yes), n (%)				7 (26.92)		8 (30.77)		11 (42.31)		*χ*^2^_2_=3.9				.14				.13		
Cannabis use (yes), n (%)				14 (50.00)		5 (17.86)		9 (32.14)		*χ*^2^_2_=2.0				.36				.09		
AUDIT^b^, mean (SD)				9.4 (5.4)		9.3 (5.8)		9.6 (5.8)		*F*_1,244_=0.08				.77				<.01		
PHQ^c^, mean (SD)				5.1(6.0)		6.0 (5.4)		5.1 (5.8)		*F*_1,244_=0.00				.99				<.01		
GAD^d^, mean (SD)				5.7 (5.5)		6.7 (5.3)		5.1 (5.3)		*F*_1,244_=0.71				.40				<.01		
Drink and drive, mean (SD)				0.3 (0.7)		0.3 (0.8)		0.3 (0.7)		*F*_1,244_=0.09				.77				<.01		
Readiness to change, n (%)				4.1 (3.0)		3.9 (2.9)		3.9 (2.9)		*F*_1,244_=0.23				.63				<.01		
Confidence reduction, n (%)				5.1 (3.0)		4.5 (2.9)		4.4 (3.3)		*F*_1,244_=2.19				.13				.01		
SIP^e^-Impulse control, n (%)				1.0 (1.5)		1.2 (1.6)		1.1(1.6)		*F*_1,244=_0.24				.63				<.01		
**Motives to drink, mean (SD)**																				
	Social			4.6 (2.3)		5.4 (2.3)		5.1 (2.4)		*F*_1,244=_2.01				.16				.01		
	Coping with anxiety			3.7 (2.1)		4.0 (2.1)		3.9 (2.3)		*F*_1,244_=0.36				.55				<.01		
	Coping with depression			4.8 (2.8)		5.3 (2.1)		5.3 (3.0)		*F*_1,244_=1.32				.25				.01		
	Enhancement			4.1 (2.3)		4.5 (2.0)		4.1 (2.0)		*F*_1,244_=0.00				.96				<.01		
	Conformity			2.6 (1.6)		2.6 (1.6)		2.7 (1.5)		*F*_1,244_=0.21				.65				<.01		
	Physical			4.4 (2.3)		4.6 (1.9)		4.5 (2.1)		*F*_1,244_=0.15				.70				<.01		

^a^Effect size reported as Cramer *V* for chi-square tests and as *R*-square for *F* tests.

^b^AUDIT: Alcohol Use Disorders Identification Test.

^c^PHQ: Patient Health Questionnaire.

^d^GAD: General Anxiety Disorder Questionnaire.

^e^SIP: Short Index of Problems.

**Table 4:** Unadjusted association between baseline characteristics and Peer Booster engagement.

Predictors					Peer Boosters																		
					0 (n=76)			1 or 2 (n=27)			3 (n=142)			Test statistic (*df*)				*P* value				Effect size^a^	
**Follow-up completion, n (%)**																							
	4 months			31 (17.42)			17 (9.55)			130 (73.03)			*χ*^2^_2_=65.6				<.001				.52		
	8 months			36 (19.57)			20 (10.87)			128 (69.57)			*χ*^2^_2_=48.4				<.001				.44		
	12 months			33 (18.97)			16(9.20)			125 (71.84)			*χ*^2^_2_=49.9				<.001				.45		
**Baseline characteristics**																							
	Sex (male), n (%)			57 (27.80)			22 (10.73)			126 (61.73)			*χ*^2^_2_=6.9				.03				.17		
	Age, mean (SD)			26.8 (6.2)			26.9 (8.1)			28.6 (6.9)			*F*_1,244_=4.08				.04				.02		
	**Race, n (%)**													*χ*^2^_2_=6.1				.05				.16	
		White	68 (32.38)			19 (9.05)			123 (58.57)														
		Other	8 (22.86)			8 (22.86)			19 (54.29)														
	Hispanic (yes), n (%)			11 (31.43)			4 (11.43)			20 (57.14)			*χ*^2^_2_=0.0				.99				.01		
**Marital status, n (%)**															*χ*^2^_6_=17.3				.008				.19
	Married			16 (25.00)			3 (4.69)			45 (70.31)													
	Living together			16 (6.53)			7 (21.88)			9 (28.13)													
	Widowed, divorced, or separated			8 (25.00)			4 (12.50)			20 (62.50)													
	Never married			36 (30.77)			13 (11.11)			68 (58.12)													
**Highest grade completed, n (%)**															*χ*^2^_4_=7.7				.10				.13
	High school or less			18 (40.91)			4 (9.09)			22 (50.00)													
	Some college			45 (31.91)			19 (13.48)			77 (54.61)													
	College or more			13 (21.67)			4 (6.67)			43 (71.67)													
**Rank, n (%)**															*χ*^2^_4_=4.2				.37				.09
	E1-E4			45 (33.09)			18 (13.24)			73 (53.68)													
	E5-E9			29 (29.90)			7 (7.22)			61 (62.89)													
	WO1-WO5/O1-O9			2 (16.67)			2 (16.67)			8 (66.67)													
Employed (yes), n (%)					69 (33.82)			19 (9.31)			116 (56.86)			*χ*^2^_2_=6.5				.04				.16	
**Household income (US $), n (%)**															*χ*^2^_6_=12.0				.06				.16
	25,000 or less			28 (37.33)			9 (12.00)			38 (50.67)													
	25,000-50,000			27 (36.00)			8 (10.67)			40 (53.33)													
	50,000 or more			18 (21.18)			7 (8.24)			60 (70.59)													
	Refused			3 (30.00)			3 (30.00)			4 (40.00)													
Ever deployed (yes), n (%)					30 (25.00)			10 (8.33)			80 (66.67)			*χ*^2^_2_=7.3				.03				.17	
Trauma exposure (yes), n (%)					26 (27.08)			10 (10.42)			60 (62.50)			*χ*^2^_2_=1.4				.50				.08	
Illicit drug use (yes), n (%)					4 (33.33)			1 (8.33)			7 (58.33)			*χ*^2^_2_=0.1				.95				.02	
Cannabis use (yes), n (%)					10 (30.30)			5 (15.15)			18 (54.55)			*χ*^2^_2_=0.6				.71				.05	
AUDIT^b^, mean (SD)					8.9 (5.1)			9.4 (5.5)			10.2 (6.1)			*F*_1,244_=2.75				.10				.01	
PHQ^c^, mean (SD)					4.4 (4.9)			6.0 (4.9)			6.2 (6.0)			*F*_1,244_=4.72				.03				.02	
GAD^d^, mean (SD)					4.8 (4.9)			6.9 (4.5)			6.7 (5.9)			*F*_1,244_=5.43				.02				.02	
Drink and drive^,^ mean (SD)					0.3 (0.6)			0.1 (0.4)			0.3 (0.7)			*F*_1,244_=0.84				.36				<.01	
Readiness to change, mean (SD)					3.9 (3.0)			3.9 (3.2)			3.5 (2.9)			*F*_1,244_=0.98				.32				<.01	
Confidence reduction, mean (SD)					4.5 (3.1)			4.4 (3.5)			4.0 (3.0)			*F*_1,244_=1.09				.30				<.01	
SIP^e^-Impulse control, mean (SD)					1.0 (1.3)			1.2 (1.2)			1.0 (1.6)			*F*_1,244_=0.24				.96				<.01	
**Motives to drink, mean (SD)**																							
	Social			5.4 (2.4)			5.3 (2.2)			5.4 (2.5)			*F*_1,244_=0.02				.88				<.01		
	Coping with anxiety			3.8 (2.1)			4.4 (2.5)			4.1 (2.2)			*F*_1,244_=1.19				.28				<.01		
	Coping with depression			5.0 (2.5)			5.8 (3.4)			5.1 (2.9)			*F*_1,244_=0.02				.89				<.01		
	Enhancement			3.8 (2.1)			5.1 (2.2)			4.3 (2.3)			*F*_1,244_=2.01				.16				.01		
	Conformity			2.6 (1.5)			2.9 (1.5)			2.8 (1.7)			*F*_1,244_=0.47				.49				<.01		
	Physical			4.5 (1.9)			4.9 (2.1)			4.6 (2.4)			*F*_1,244_=0.04				.83				<.01		

^a^Effect size reported as Cramer *V* for chi-square tests and as *R*-square for *F* tests.

^b^AUDIT: Alcohol Use Disorders Identification Test.

^c^PHQ: Patient Health Questionnaire.

^d^GAD: General Anxiety Disorder Questionnaire.

^e^SIP: Short Index of Problems.

### Adjusted Outcomes

Table 5 presents the results of the 3 multinomial logistic models of the candidate predictors of booster session engagement. A Web Booster model, Peer Booster model, and Combined model with treatment arm (Web or Peer) entered as a candidate predictor of the number of booster sessions completed (0, 1, 2, or 3) are presented. All models used a stepwise variable selection routine to identify predictors of engagement where candidate predictors were entered sequentially, and variables were retained at either step if they were below the threshold of *P*≤.30 level. The Web Booster model retained 9 candidate predictors of booster engagement: gender, education level, rank, being deployed, having experienced trauma, anxiety, frequency of alcohol use, social motives for drinking, and income. In the final Web Booster model, participants who had completed high school or lower (adjusted odds ratio [aOR] 0.21, 95% CI 0.07-0.64; *P*=.006) and those who had completed any college-level education (aOR 0.39, 95% CI 0.16-0.93, *P*=.03) were significantly less likely to complete all three boosters when compared with those with higher education (Table 5 for summary of models; Multimedia Appendix 2 for all adjusted odds ratios of model predictors).

**Table 5:** Model-adjusted stepwise models of predictors of engagement in Combined, Web, and Peer Booster arms.

Selected candidate predictors	Combined		Web Boosters	Peer Boosters		
	χ^2^ (*df*)	*P* value	χ^2^ (*df*)	*P* value	χ^2^ (*df*)	*P* value
Treatment arm	*10.0 (2)* ^a^	*.01*	NA^b^	NA	NA	NA
Education	*17.9 (4)*	*.001*	*9.6 (4)*	*.047*	8.7 (4)	.07
Income	*12.5 (4)*	.*02*	5.9 (4)	.20	—^c^	—
Employed	3.3 (2)	.19	—	—	*6.2 (2)*	.*04*
Rank	*6.0 (2)*	*.049*	*10.5 (2)*	*.005*	—	—
Anxiety (GAD^d^)	4.6 (2)	.10	3.7 (2)	.16	—	—
Alcohol use severity (AUDIT^e^)	2.6 (2)	.27	—	—	—	—
Confidence can reduce alcohol use	3.5 (2)	.18	—	—	—	—
Gender	—	—	4.2 (2)	.12	*8.2 (2)*	*.02*
Motive: social	—	—	3.4 (2)	.18	3.6 (2)	.16
Deployed	—	—	3.9 (2)	.14	—	—
Binge drinking frequency	—	—	4.1 (2)	.13	—	—
Traumatic event	—	—	5.7 (2)	.06	—	—
Marital status	—	—	—	—	*13.4 (6)*	*.02*
Depression (PHQ^f^)	—	—	—	—	*6.0 (2)*	*.049*
Motive: enhancement	—	—	—	—	*10.1 (2)*	*.01*
Motive: coping with depression	—	—	—	—	2.9 (2)	.23
Drink and drive	—	—	—	—	3.8 (2)	.15

^a^Italicized values indicate statistical significance at the α<.05 threshold.

^b^NA indicates that the predictor (ie, treatment arm) was not included as a candidate predictor in the model. All variables selected in each model were reported.

^c^—: Indicates that candidate variable was not retained in the model.

^d^GAD: General Anxiety Disorder Questionnaire.

^e^AUDIT: Alcohol Use Disorders Identification Test.

^f^PHQ: Patient Health Questionnaire.

The Peer Booster model retained 9 predictors: employment status, marital status, gender, education level, enhancement, social, and coping with depression motives for drinking, drinking while driving, and depression symptoms. Those who were employed were significantly less likely to complete 1 or 2 boosters than those who were unemployed (aOR 0.19, 95% CI 0.05-0.71; *P*=.01), although this relationship was not statistically significant for engagement in all three boosters (aOR 0.40, 95% CI 0.14-1.11; *P*=.08). Men were significantly more likely to complete all 3 boosters than women (aOR 3.53, 95% CI 1.47-8.48 *P*=.005). Those who were living together were significantly less likely to complete all three booster sessions than those who were married (aOR 0.23, 95% CI 0.08-0.68; *P*=.008). Those who were depressed were significantly more likely to complete all three boosters (aOR 1.11, 95% CI 1.02-1.20; *P*=.01). Finally, those who reported drinking to enhance positive feelings were significantly more likely to complete 1 or 2 booster sessions (aOR 1.59, 95% CI 1.18-2.16; *P*=.003) and complete all 3 sessions (aOR 1.27, 95% CI 1.04-1.56; *P*=.02) than people with lower enhancement motives (Multimedia Appendix 3 for all adjusted odds ratios of model predictors).

The combined model retained 8 of the candidate predictors of booster engagement: treatment arm (Web or Peer), education level, income, military rank, anxiety, confidence in ability to reduce alcohol use, and alcohol use severity. Treatment arm, education, income, and rank were statistically significant in the final model. People randomized to the Peer Booster arm were more likely to engage in booster sessions overall; however, neither contrast between some sessions (aOR 0.63, 95% CI 0.34-1.15; *P*=.13) nor all sessions (aOR 1.51, 95% CI 0.99-2.32; *P*=.06) was statistically significant. Those who had completed high school or lower (aOR 0.24, 95% CI 0.12-0.47; *P*<.001) and those who had completed some college (aOR 0.44, 95% CI 0.25-0.76; *P*=.004) were significantly less likely than those who had completed college to complete all boosters. Those who reported incomes greater than US $50,000 were more likely to complete all boosters than those who made US $25,001 to US $50,000 (aOR 2.11, 95% CI 1.23-3.63; *P*=.007) and to complete 1 or 2 boosters than no boosters (aOR 2.19, 95% CI 1.03-4.70; *P*=.04). Finally, those of lower rank were more likely to complete 1 or 2 boosters than no boosters (aOR 2.09, 95% CI 1.05-4.15; *P*=.03; Multimedia Appendix 4 for all adjusted odds ratios of model predictors).

## Discussion

### Principal Findings

This study provides novel findings regarding engagement in boosters delivered through the web or peer modality. Results showed that 65.2% (320/491) of participants attended at least 1 booster session, most people who attended one booster session attended all three boosters, and engagement was higher when boosters were delivered by peers than via an interactive website. Previous analyses of engagement in boosters have generally used emergency room populations and have reported difficulty maintaining engagement after the initial BIs, in part because of low rates of cell phone ownership and high rates of homelessness [25,35]. In contrast, our sample of adults actively enrolled in the National Guard is different in that nearly all participants had access to a phone or had a home address, all had income from their guard duties, and most were also employed in their civilian role. The Peer Booster delivery format was more successful in fostering engagement in the booster sessions. This may be related to the effect of human-to-human peer interactions. Peer Boosters may have been further enhanced compared with simple person-delivered boosters since military veterans conducted Peer Booster sessions. There is generally a strong sense of camaraderie between those active in the reserve components, most of whom are also officially veterans and have previously served in the active military. The percentage of reserve component members who have official veteran status has markedly increased in the past 15 years since reserve component members have been activated for overseas deployment in the ongoing wars in the Middle East. It is also notable that once the initial contact was made with the peer during the first booster session, a higher percentage of these participants (142/169, 84%) completed all 3 boosters than was the case with the Web Booster sessions (108/151, 71.5%; despite the fact that it was interactive and tailored; χ^2^_1_=7.3; *P*=.007). This finding is a potential additional indicator that peer alliances drove continued booster engagement. It has been suggested that the characteristics of the person delivering the booster may be important. For example, Longabuagh et al [25] reported on the wide variation of success among their in-person boosters in getting participants to do a single booster in their study and recommended more studies on the characteristics of the persons delivering the boosters.

Future research should identify whether general factors related to human interaction as opposed to military-specific factors such as camaraderie or other characteristics of similarity between service members were the primary drivers of greater engagement in the Peer compared with Web Booster sessions.

Research on web- and app-based BIs is increasing because of their potential cost effectiveness and scalability to increase the reach of interventions for those historically underserved through traditional health models of health care. Similarly, there is a need to assess the possible combination of web- and peer-based delivery systems for boosters following BIs. The case may be that combining web- with person-based delivery can be optimized to balance cost and scalability benefits of web-delivered boosters with increased engagement from person-delivered boosters. This balance can take into account the inherent increased cost of a peer delivery format, where a staff member must be paid and where the length of the booster session may be longer because the peer may spend time, particularly initially, building a relationship of trust and respect with the participant.

Few previous studies, which included boosters following a web-delivered BI, have examined the predictors of booster engagement. Longabaugh et al [25] reported that for an alcohol misuse BI presented in person in the emergency room, with the booster also in person, 69% of participants completed the booster, and those who completed the booster had reduced alcohol-related consequences and injuries, whereas those without the booster did not. Hatch-Maillette et al [35] reported details of booster engagement for a drug misuse BI presented in person in an emergency department followed by two in-person booster sessions; 57.3% did the first booster and 39.1% completed both boosters. Our findings fit within the context of previous studies. The Peer Booster group engaged in a single booster session at a rate comparable with those reported previously [25] and completed all 3 boosters at a much higher rate than in the study by Hatch-Maillete et al [35]. The Web Booster participants performed only moderately worse than those in the Longabaugh study and slightly better in completing the full course of boosters than participants in the study by Hatch-Maillette et al [35]. These findings suggest that largely phone-based Peer Boosters may increase booster engagement compared with in-person booster delivery, and that web-delivered boosters may be associated with similar engagement as in-person boosters.

A number of factors potentially affect whether a booster is optimally impactful, including the content of the booster, the dosage of the booster delivered, the timing of the booster following the BI, and whether the booster is sufficient to motivate the participant to continue to engage. When considering wide-scale dissemination, delivering boosters in person is expensive compared with web or phone delivery options. The cost effectiveness of booster delivery could be potentially addressed by using multiple options for delivery of boosters and identifying subsets of individuals who require more labor-intensive and expensive personal delivery approaches. VA has invested heavily in hiring peer counselors. Nevertheless, the number of counselors is likely to be insufficient to meet the needs of all patients. VA has begun to assess whether peer counselors can be more effective than web- or app-based eHealth interventions when integrated into primary care clinics [41]. A natural extension of this work is to identify additional settings, such as armories, to integrate peer counselors to connect with service members and provide ongoing support. The military mental health and resilience building systems also use fellow enlisted and noncommissioned members as health technicians. In both systems, it would be useful to be able to identify subsets of veterans and military members who would most benefit from eHealth interventions that include a peer support component.

In this reserve component military population, the combined model indicates that those with relatively higher socioeconomic status engaged more often in boosters. Those with higher rank, higher income, and more education all engaged more. All three of these characteristics were intercorrelated in the military population. The results from the separate models for web and peer delivery modes suggest that participant characteristics had differential effects on booster engagement.

The finding that participants who report more depression are more likely to engage in boosters in the Peer Booster arm is notable given the high prevalence of mental health comorbidities in people who misuse alcohol. Not surprisingly, depression symptoms were high in this alcohol-misusing sample. People experiencing depression represent a considerable and clinically meaningful subset of the total study population and could be a meaningful target for identifying those service members that may improve booster engagement if provided with a peer delivery format.

Web Boosters have many practical advantages. It is noteworthy that older participants were more likely to complete Web Boosters. Although one might expect younger participants to be more drawn to computer interaction, our sample was somewhat age restricted, reflecting the age demographics of the military, including the guard. The constellation of variables predicting booster engagement in the web format (higher rank, higher socioeconomic status, higher age) suggests that whatever greater affinity younger participants might have for computer interaction, it is likely to be secondary to the tendency of older, higher ranked individuals with better socioeconomic status who may be more willing to adhere to the intervention.

### Limitations

These findings should be considered in the context of several limitations. The nature of the sample (members of the National Guard of a single state) potentially limits the generalizability of the results. Although we screened a substantial proportion of the total population of the Guard members in Michigan (about one-third), randomly selected the units to assure broad representation of all military occupational categories (eg, infantry, logistics, military police) in the sample, and the Michigan National Guard is largely comparable with the National Guard nationally, future work is needed to identify the generalizability of booster engagement in other National Guard populations. Women were underrepresented based on the general population, but adequately represented relative to their prevalence in the Michigan National Guard. This is generally consistent with military populations, although the percentages of women in the military, and in most occupational categories within the military, are increasing [61]. The study was not designed to be powered to detect head-to-head differences between the 2 booster delivery formats. These findings are exploratory in nature and require further investigation in studies powered to detect key indicators of booster engagement across delivery modalities.

In summary, the use of telephone or in-person peer support to provide encouragement and lived experience in helping individuals make behavioral changes has been successfully employed in a number of settings. The inclusion of peers to provide booster follow-ups with National Guard members experiencing issues related to alcohol use promotes engagement and fits well with the goals of this and other organizations that work toward maintaining and improving the health of their members so that they do well at work and in their personal lives. These exploratory analyses suggest promising directions for future research aimed at identifying subsets of individuals who require more intensive booster delivery modalities, such as peer delivery, to optimally facilitate engagement. Additional work to replicate the predictors of booster engagement noted here, in addition to considering other possible indicators of engagement, are needed. In addition, exploration of combining web-delivered boosters with some person-delivered components of booster sessions may balance pragmatic concerns around cost and scalability with the need to foster booster engagement, especially among at-risk subsets of service members who misuse alcohol.

## Appendix

Multimedia Appendix 1 Mission Strong Consort Diagram for participants that misuse alcohol.

Multimedia Appendix 2 Model-adjusted odds ratios and 95% confidence limits for web-delivered booster condition.

Multimedia Appendix 3 Model-adjusted odds ratios and 95% confidence limits for peer-delivered booster condition.

Multimedia Appendix 4 Model-adjusted odds ratios and 95% confidence limits for both booster conditions.

## Abbreviations

aOR: adjusted odds ratio
BI: brief intervention
EUC: enhanced usual care
GAD: General Anxiety Disorder Questionnaire
GLM: General linear modeling
MINI: Mini International Neuropsychiatric Interview
PHQ-9: Patient Health Questionnaire
SIP: Short Index of Problems
