# Supplementary material for: Predictors of Booster Engagement Following a Web-Based Brief Intervention for Alcohol Misuse Among National Guard Members: Secondary Analysis of a Randomized Controlled Trial
Source: JMIR Ment Health. 2021 Oct 26;8(10):e29397. doi: 10.2196/29397 (PMC8579213; doi:10.2196/29397)
Supplement: Multimedia Appendix 1 [file mental_v8i10e29397_app1.docx]

Figure 1. Mission Strong Consort Diagram for participants that misuse alcohol.


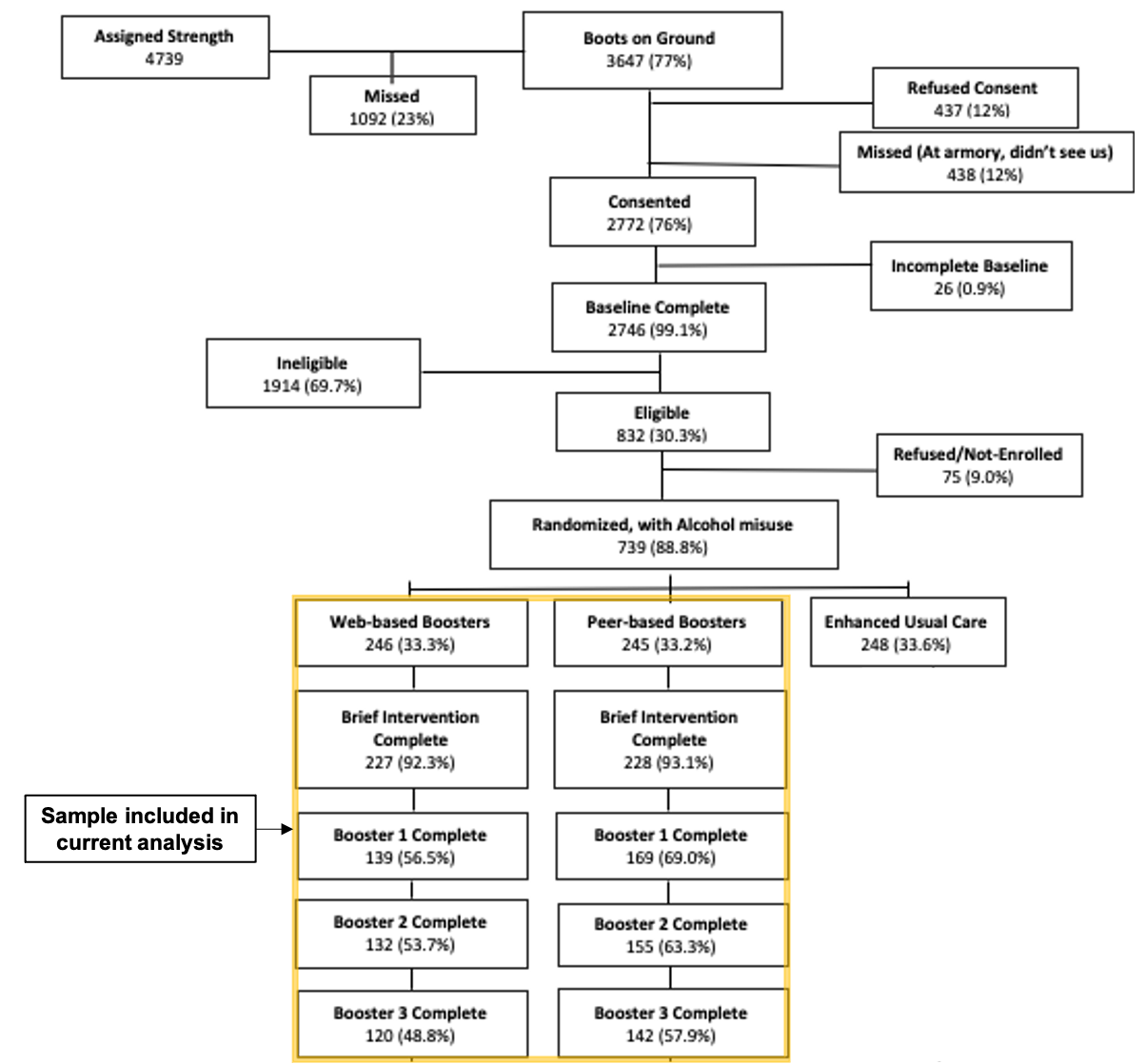


Only those participants who were randomized to either the web-based or peer-based treatment arms were included in this study aimed at looking at booster session engagement.
