# Supplementary material for: Predictors of Booster Engagement Following a Web-Based Brief Intervention for Alcohol Misuse Among National Guard Members: Secondary Analysis of a Randomized Controlled Trial
Source: JMIR Ment Health. 2021 Oct 26;8(10):e29397. doi: 10.2196/29397 (PMC8579213; doi:10.2196/29397)
Supplement: Multimedia Appendix 2 [file mental_v8i10e29397_app2.docx]

Table 1. Model-adjusted odds ratios and 95% confidence limits for web-delivered booster condition

|  |  |  |  |
| --- | --- | --- | --- |
| **Characteristics** | **Boosters Completed** Reference: no boosters | **Adjusted Odds Ratio** | **95% Confidence Limit** |
| Gender  Reference: Female | 1 or 2 boosters | 0.42 | 0.13-1.32 |
|  | 3 boosters | 0.37 | 0.14-0.98 |
| Education  Highschool or less compared to college or more | 1 or 2 boosters | 0.88 | 0.23-3.42 |
|  | 3 boosters | 0.21 | 0.07-0.64 |
| Education  Some college compared to college or more | 1 or 2 boosters | 0.94 | 0.30-2.93 |
|  | 3 boosters | 0.39 | 0.16-0.93 |
| Income Less than $25,000 compared to $25,001-$50K | 1 or 2 boosters | 0.83 | 0.28-2.45 |
|  | 3 boosters | 1.08 | 0.46-2.53 |
| Income $50,000 or more compared to $25,001-$50K | 1 or 2 boosters | 2.52 | 0.92-6.90 |
|  | 3 boosters | 2.02 | 0.91-4.49 |
| Rank  Reference: Not E1-E4 | 1 or 2 boosters | 2.70 | 0.96-7.57 |
|  | 3 boosters | 0.52 | 0.24-1.15 |
| Deployment Reference: Never deployed | 1 or 2 boosters | 1.09 | 0.44-2.75 |
|  | 3 boosters | 0.52 | 0.24-1.10 |
| Trauma Reference: No reported trauma | 1 or 2 boosters | 3.31 | 1.19-9.18 |
|  | 3 boosters | 2.04 | 0.89-4.66 |
| Anxiety (GAD) | 1 or 2 boosters | 0.99 | 0.91-1.08 |
|  | 3 boosters | 0.94 | 0.88-1.01 |
| Binge drinking frequency | 1 or 2 boosters | 0.91 | 0.83-1.00 |
|  | 3 boosters | 1.00 | 0.95-1.06 |
| Motive: Social | 1 or 2 boosters | 1.20 | 0.99-1.46 |
|  | 3 boosters | 1.09 | 0.93-1.27 |
