# Supplementary material for: Predictors of Booster Engagement Following a Web-Based Brief Intervention for Alcohol Misuse Among National Guard Members: Secondary Analysis of a Randomized Controlled Trial
Source: JMIR Ment Health. 2021 Oct 26;8(10):e29397. doi: 10.2196/29397 (PMC8579213; doi:10.2196/29397)
Supplement: Multimedia Appendix 3 [file mental_v8i10e29397_app3.docx]

Table 2. Model-adjusted odds ratios and 95% confidence limits for peer-delivered booster condition

|  |  |  |  |
| --- | --- | --- | --- |
| **Characteristics** | **Boosters Completed** Reference: no boosters | **Adjusted Odds Ratio** | **95% Confidence Limit** |
| Gender  Reference: Female | 1 or 2 boosters | 2.88 | 0.74-11.21 |
|  | 3 boosters | 3.53 | 1.47-8.48 |
| Marital status Living together compared to married | 1 or 2 boosters | 2.57 | 0.47-14.03 |
|  | 3 boosters | 0.23 | 0.08-0.68 |
| Marital status Never married compared to married | 1 or 2 boosters | 1.44 | 0.31-6.64 |
|  | 3 boosters | 0.72 | 0.33-1.56 |
| Marital status Widowed, separated or divorced compared to married | 1 or 2 boosters | 3.65 | 0.57-23.33 |
|  | 3 boosters | 0.97 | 0.34-2.77 |
| Education  Highschool or less compared to college or more | 1 or 2 boosters | 0.29 | 0.05-1.88 |
|  | 3 boosters | 0.32 | 0.12-0.87 |
| Education  Some college compared to college or more | 1 or 2 boosters | 1.28 | 0.32-5.08 |
|  | 3 boosters | 0.50 | 0.23-1.11 |
| Employment status Reference: not employed | 1 or 2 boosters | 0.19 | 0.05-0.71 |
|  | 3 boosters | 0.40 | 0.14-1.11 |
| Depression (PHQ) | 1 or 2 boosters | 1.07 | 0.94-1.21 |
|  | 3 boosters | 1.11 | 1.02-1.20 |
| Drink and drive | 1 or 2 boosters | 0.21 | 0.04-1.01 |
|  | 3 boosters | 0.94 | 0.55-1.61 |
| Motive: Social | 1 or 2 boosters | 0.79 | 0.61-1.03 |
|  | 3 boosters | 0.89 | 0.76-1.04 |
| Motive: Enhancement | 1 or 2 boosters | 1.59 | 1.18-2.16 |
|  | 3 boosters | 1.27 | 1.04-1.56 |
