# Supplementary material for: Predictors of Booster Engagement Following a Web-Based Brief Intervention for Alcohol Misuse Among National Guard Members: Secondary Analysis of a Randomized Controlled Trial
Source: JMIR Ment Health. 2021 Oct 26;8(10):e29397. doi: 10.2196/29397 (PMC8579213; doi:10.2196/29397)
Supplement: Multimedia Appendix 4 [file mental_v8i10e29397_app4.docx]

Table 3. Model-adjusted odds ratios and 95% confidence limits for both booster conditions

| **Characteristics** | **Boosters Completed** Reference: no boosters | **Adjusted Odds Ratio** | **95% Confidence Limit** |
| --- | --- | --- | --- |
| Arm  Reference: Web-delivered | 1 or 2 boosters | 0.63 | 0.34-1.15 |
|  | 3 boosters | 1.51 | 0.99-2.32 |
| Education  Highschool or less compared to college or more | 1 or 2 boosters | 0.43 | 0.16-1.16 |
|  | 3 boosters | 0.24 | 0.12-0.47 |
| Education  Some college compared to college or more | 1 or 2 boosters | 0.76 | 0.34-1.71 |
|  | 3 boosters | 0.44 | 0.25-0.76 |
| Income Less than $25,000 compared to $25,001-$50K | 1 or 2 boosters | 0.80 | 0.38-1.71 |
|  | 3 boosters | 0.88 | 0.52-1.49 |
| Income $50,000 or more compared to $25,001-$50K | 1 or 2 boosters | 2.19 | 1.03-4.70 |
|  | 3 boosters | 2.11 | 1.23-3.63 |
| Employment status Reference: Not employed | 1 or 2 boosters | 0.49 | 0.20-1.17 |
|  | 3 boosters | 0.60 | 0.31-1.16 |
| Rank Reference: E1-E4 | 1 or 2 boosters | 2.09 | 1.05-4.15 |
|  | 3 boosters | 0.94 | 0.59-1.49 |
| Anxiety (GAD) | 1 or 2 boosters | 1.07 | 1.00-1.13 |
|  | 3 boosters | 1.01 | 0.97-1.06 |
| Alcohol use severity (AUDIT) | 1 or 2 boosters | 0.98 | 0.92-1.04 |
|  | 3 boosters | 1.02 | 0.98-1.07 |
| Confidence can reduce alcohol use | 1 or 2 boosters | 0.94 | 0.86-1.03 |
|  | 3 boosters | 0.94 | 0.88-1.01 |
